# Effect of neurofilament analysis on the diagnostic delay in amyotrophic lateral sclerosis

**DOI:** 10.1111/cns.13960

**Published:** 2022-09-01

**Authors:** Maxim De Schaepdryver, Pegah Masrori, Philip Van Damme, Koen Poesen

**Affiliations:** ^1^ Laboratory for Molecular Neurobiomarker Research, Department of Neurosciences Leuven Brain Institute, KU Leuven Leuven Belgium; ^2^ Laboratory of Neurobiology Center for Brain & Disease Research, VIB Leuven Belgium; ^3^ Department of Neurology University Hospitals Leuven Leuven Belgium; ^4^ Experimental Neurology, Department of Neurosciences Leuven Brain Institute, KU Leuven Leuven Belgium; ^5^ Laboratory Medicine University Hospitals Leuven Leuven Belgium

**Keywords:** amyotrophic lateral sclerosis, cerebrospinal fluid, diagnostic delay, neurofilament

## Abstract

**Aims:**

The aim of this study was to investigate whether neurofilament light (NfL) and phosphorylated neurofilament heavy (pNfH) in cerebrospinal fluid (CSF), sampled prior to referral to a neuromuscular reference center (NMRC), shorten the diagnostic delay in patients with amyotrophic lateral sclerosis.

**Methods:**

In this retrospective study, patients with ALS were included with (i) determination of neurofilaments (Nfs) before referral to the NMRC (preC‐Nfs ALS, *n* = 58), (ii) determination of Nfs at the NMRC (C‐Nfs, *n* = 54) or (iii) with no determination of Nfs (C‐No Nfs, *n* = 180). Fifty‐six disease controls were included.

**Results:**

The preC‐Nfs cohort had CSF sampled 2.2 months (range: 0.6–12.0 months) before referral to the NMRC. In this cohort, the diagnostic delay was significantly shorter [median (range): 8.24 (2.37–49.7) months] than in the C‐Nfs cases [median (range): 11.4 (2.93–86.5) months; *p* < 0.05], but not in the C‐No Nfs cases. When including the disease progression rate and the presence of a genetic mutation as covariates, the difference ceased to exist (*p* = 0.14). pNfH and NfL levels in the preC‐Nfs cohort were significantly higher than in disease controls (*p* < 0.0001). Both Nfs showed a similar discriminating performance.

**Conclusions:**

CSF Nfs assessed before the diagnosis of ALS at a NMRC decreased the diagnostic delay in specific cases by 3 months and only when other covariates were not taken into account.

## Graphical abstract

Neurofilaments assessed in cerebrospinal fluid before diagnosis of ALS at the neuromuscular reference center decreased the diagnostic delay by 3 months only when other covariates like disease progression rate and the presence of a genetic mutation were not considered.

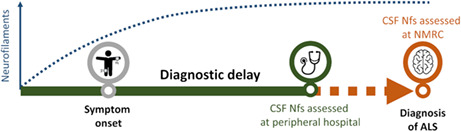

## 1 INTRODUCTION

Amyotrophic lateral sclerosis (ALS) is a progressive disease characterized by the degeneration of both upper and lower motor neurons.^1^ Approximately 10% of patients inherit the disease, while others are classified as sporadic ALS.^2^ To date, ALS remains a fatal disease as no cure is available. The median survival after symptom onset is 20 to 48 months.^3^ Available therapies like riluzole and edaravone have only a modest effect on the disease progression and survival.^4^, ^5^, ^6^ Notwithstanding the advances in understanding the disease pathophysiology, the development of therapies significantly affecting the survival in patients with ALS is limited. A contributing factor is the diagnostic delay, which is the time from symptom onset until diagnosis at a neuromuscular reference center (NMRC), or a tertiary ALS referral center.^7^, ^8^ In most clinics worldwide, the median diagnostic delay is about 10–12 months.^8^, ^9^, ^10^, ^11^, ^12^, ^13^ This delay, which accounts for a significant period of the total survival in patients with ALS, impedes to include patients in clinical trials in the early stage of the disease. This stage, however, is believed to be the most opportune window to therapeutically slow down the disease progression.

These observations highlight the importance of biomarkers for an early and correct diagnosis of ALS. Fluid biomarkers can be useful tools for clinicians in the diagnostic work‐up of patients with ALS. Neurofilaments have shown great potential as biomarkers in many neurodegenerative diseases including ALS.^14^, ^15^ These intermediate class IV filaments are essential for neuronal growth and structural stability of large, myelinated axons.^16^ We and others have shown that the neurofilament light chain (NfL) and phosphorylated neurofilament heavy chain (pNfH) are significantly higher in the cerebrospinal fluid (CSF) of patients with ALS, as assessed at a NMRC, than in disease mimics.^17^, ^18^, ^19^ Early evidence now demonstrates that CSF NfL levels are elevated 1 year before symptom onset in patients who are genetically at risk for ALS.^20^, ^21^ In addition, we showed that pNfH levels in serum are increased in almost 60% of patients with ALS before diagnosis at a NMRC.^22^

In this study, we now explore as primary objective if neurofilaments in CSF sampled before referral for diagnosis to a NMRC affect the diagnostic delay in ALS, and as a secondary objective whether early stage determined neurofilaments have a similar diagnostic performance as compared to CSF sampled at a NMRC.

## 2 MATERIALS AND METHODS

In total, 292 patients with ALS, all diagnosed at the NMRC of the University Hospitals Leuven according to the revised El Escorial and Awaji criteria, were included between March 2017 and June 2021 in this observational retrospective study.^23^ Patients with ALS were divided into three cohorts: (i) 58 patients with ALS who had CSF sampled by a neurologist at a regional hospital to have neurofilament assessed before referral to the NMRC (preC‐Nfs), (ii) 54 patients with ALS who had CSF sampled at the NMRC for neurofilament assessment (C‐Nfs), and (iii) 180 patients who were diagnosed at the NMRC without any neurofilament assessment (C‐No Nfs). The revised ALS functional rating scale (ALSFRS‐R) score was obtained at time of diagnosis for all three ALS cohorts. The disease duration was defined as time from symptom onset until sampling. Gene testing for *C9orf72*, *SOD1*, *TARDBP*, and *FUS* was proposed for patients with ALS using TP‐PCR (*C9orf72*) or direct sequencing (all other genes).

A disease control cohort of 56 patients was consecutively included between February 2017 and December 2020 by local neurologists at regional hospitals. Neurofilaments were requested by the local neurologist for diagnostic purposes, mainly when there was a suspicion of a motor neuron disease. The final diagnoses of the control cohort were obtained through review of the patient's electronic medical records by a neurologist (PM) affiliated to the NMRC of the University Hospitals Leuven (Table S1). Five control patients were excluded as no definite diagnosis was given to the patient.

All CSF samples collected by local neurologists were transferred at room temperature to the laboratory medicine department at the University Hospitals Leuven within 5 days after lumbar puncture. Subsequently, samples were divided into aliquots of 1 ml and stored at −80°C until analysis. CSF samples collected at the NMRC were divided into aliquots of 1 ml and stored at −80°C within 24 h after sampling. All CSF samples were consecutively collected and were not included in any previous publications. Commercially available IVD‐labeled ELISAs were used according to the manufacturer's protocol for measuring NfL (10–7001 CE, Umandiagnostics AB, Umea, Sweden) and pNfH (Equation 6561–9601, Euroimmun AG, Lübeck, Germany). All assays were then performed at the laboratory of molecular neurobiomarker research (KU Leuven) by the same operator (MDS). A low and high internal quality control sample was measured in each and every run for pNfH and NfL. The intra‐assay variability and inter‐assay variability were <6% and <11% for both biomarkers, respectively. The study was approved by the ethical committee of the University Hospitals Leuven (S50354 and S60768), and written informed consent was waived.

Shapiro–Wilk test was used for assessing normality of data. Group comparisons were performed with a Mann–Whitney *U*‐test or a Kruskal–Wallis test corrected for multiple comparison with Dunn's post hoc test. Correlations between continuous variables were tested with Spearman's rank test. The disease progression rate was calculated by the difference between the ALSFRS‐R score at time of diagnosis and 48, divided by the number of months between onset and time of ALSFRS‐R assessment. The diagnostic delay and the disease progression rate were log‐transformed to achieve a normal distribution before entering the parameters in an ANCOVA model. Receiver operating characteristic curve analysis was performed to establish the diagnostic accuracy, sensitivity, and specificity of each biomarker. The cutoff corresponding to the highest Youden index was selected. A logistic regression was used to calculate odds ratios providing a relationship between the neurofilament levels and the probability of the disease. GraphPad Prism (V.9.1.2; GraphPad Software, San Diego, California, USA), MedCalc (V.18.11.3; MedCalc Software bvba, Ostend, Belgium), and SPSS (V.27; IBM Corporation, Chicago, IL, USA) were used.

## 3 RESULTS

### 3.1 The effect of early disease stage determined neurofilaments on the diagnostic delay

Patient demographics of the three ALS cohorts and disease control cohort are presented in Table 1. No significant differences in age or gender were observed among all groups. A concomitant diagnosis of frontotemporal dementia (FTD) was present in 5 (8.6%), 4 (7.4%), and 15 (8.3%) patients in the preC‐Nfs, the C‐Nfs, and the C‐No Nfs cohort, respectively. In addition, phenotypical variants like the flail arm, upper or lower motor neuron predominance, and axial variant were equally present in the three ALS cohorts (Table 1). At time of diagnosis at the NMRC, no significant difference in the distribution of the diagnostic certainty was observed between the ALS cohorts (Table 1). The median ALSFRS‐R score at time of diagnosis and the presence of bulbar onset were not different between the three ALS cohorts (Table 1). A genetic mutation was detected in 5 (10%), 10 (19%), and 25 (19%) patients of the preC‐Nfs, the C‐Nfs, and the C‐No Nfs cohort, respectively (Table S2). In a subanalysis, riluzole therapy was initiated at a later disease stage in the C‐Nfs cohort as compared to the preC‐Nfs and C‐No Nfs cohort (*χ* ^2^ = 28.8, *p* < 0.0001, Table 1).

**TABLE 1:** Patient demographics and neurofilament levels.

	Amyotrophic lateral sclerosis			Disease controls
	preC‐Nfs	C‐Nfs	C‐No Nfs	
N	58	54	180	56
CSF pNfH (pg/ml)	2144 (180–6310)	2034 (126–12,568)	–	376 (48–13,478)
CSF NfL (pg/ml)	5493 (655–27,284)	4103 (605–35,344)	–	1025 (193–15,738)
Gender (*n* of m/f, %)	33/25 (57/43%)	29/25 (54/46%)	114/66 (63/37%)	40/16 (71/29%)
Age at onset (years)^a^	65 (30–83)	64 (32–81)	63 (25–86)	63 (18–87)
Bulbar onset (*n*, %)	21 (36%)	16 (30%)	49 (27%)	–
ALSFRS‐R (points)	40 (14–48)	39 (8–48)	40 (10–47)	–
Disease progression rate (points/month)	0.79 (0.00–9.40)	0.71 (0.05–3.50)	0.80 (0.00–5.14)	–
Clinical phenotypes				
Axial variant (*n*, %)	2 (4%)	–	2 (1%)	–
UMN predominant (*n*, %)	2 (4%)	3 (6%)	5 (3%)	–
LMN predominant (*n*, %)	1 (2%)	2 (4%)	2 (1%)	–
Flail arm (*n*, %)	–	1 (2%)	–	–
Riluzole started^b^				
Before NMRC visit (*n*, %)	24 (46%)	2 (5%)	27 (57%)	
After/At NMRC visit (*n*, %)	28 (54%)	40 (95%)	20 (43%)	
El Escorial category^c^				
Possible ALS	18 (31%)	26 (48%)	61 (34%)	–
Probable ALS	34 (59%)	27 (50%)	101 (56%)	–
Definite ALS	6 (10%)	1 (2%)	18 (10%)	–

*Note*: Median and range are given.

Abbreviations: C‐Nfs, CSF sampling and neurofilament assessment at the NMRC by experienced neurologists; C‐No Nfs, diagnosed at the NMRC without a neurofilament assessment; CSF, cerebrospinal fluid; NfL, neurofilament light; pNfH, phosphorylated neurofilament heavy; preC‐Nfs, CSF sampling and neurofilament assessment by local neurologists at regional hospitals before referral to the NMRC (neuromuscular reference center).

^a^ Age at sampling is given for the disease controls.

^b^ Subanalysis included 52 preC‐Nfs, 42 C‐Nfs, and 47 C‐No Nfs patients.

^c^ Suspected ALS was combined with possible ALS, and probable ALS was merged with probable ALS lab supported.

**FIGURE 1:**
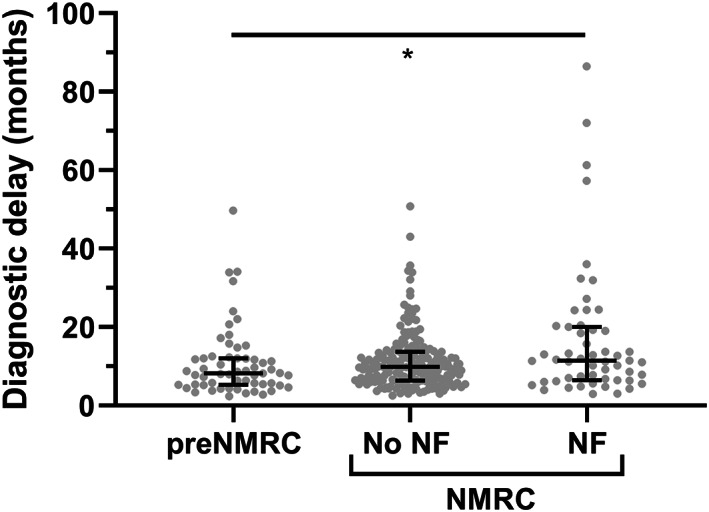
Influence of neurofilament determination on the diagnostic delay in ALS. Scatter plot showing the diagnostic delay for patients with amyotrophic lateral sclerosis (ALS) with neurofilament determination before referral to the neuromuscular reference center (preC‐Nfs) and patients with ALS with Nfs (C‐Nfs) or without Nfs (C‐No Nfs) determination at the neuromuscular reference center. Kruskal–Wallis test with Dunn's post hoc test, *p < 0.05. Median and interquartile range are presented on top of the plot.

**FIGURE 2:**
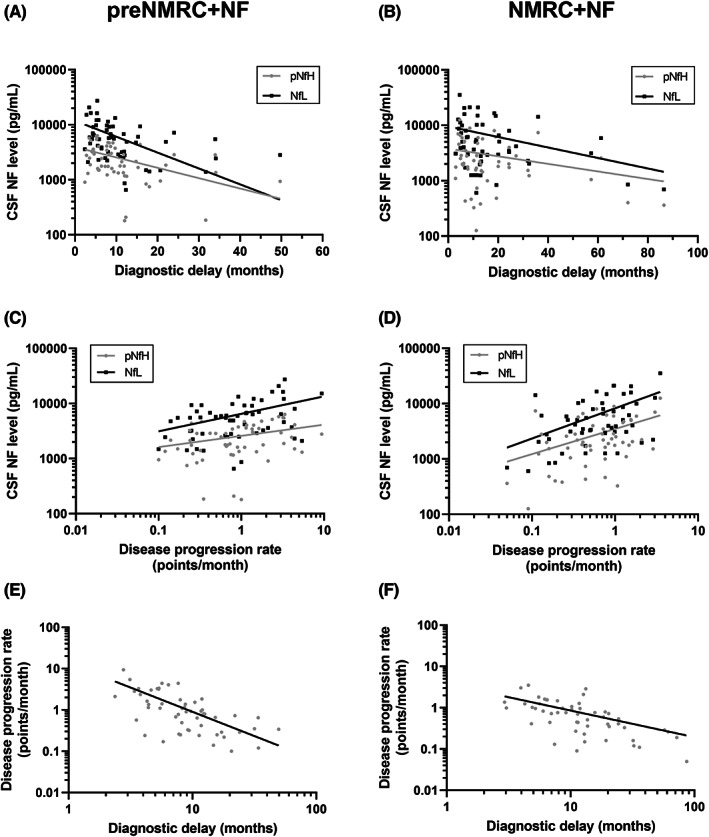
Correlations between disease progression rate, diagnostic delay, and neurofilaments. Correlations assessed in patients with amyotrophic lateral sclerosis (ALS) with neurofilament (NF) determination before referral to the neuromuscular reference center (preC‐Nfs) (A, C) and in patients with ALS at the neuromuscular reference center (C‐Nfs) (B, D). Phosphorylated neurofilament heavy (pNfH) and neurofilament light (NfL) levels in cerebrospinal fluid (CSF) correlated with diagnostic delay (A, B) and disease progression rate (C, D). Correlation between disease progression rate and diagnostic delay (E, F). Spearman's rank correlation was performed.

The disease duration at sampling in the preC‐Nfs cohort (median: 6.15 months, range: −1.57 to 47.3) was significantly shorter than in the C‐Nfs cohort (median: 11.6 months, range: 2.60–84.7 months; *p* < 0.0001). The samples of the preC‐Nfs cohort were collected with a median of 2.2 months (range: 0.6–12.0 months) before diagnosis at the NMRC. The diagnostic delay in the preC‐Nfs cohort was significantly shorter (median: 8.24 months, range: 2.37–49.7 months) in comparison with the C‐Nfs cohort (median: 11.4 months, range: 2.93–86.5 months; *p* < 0.05), but not when compared to the C‐No Nfs cohort (median: 9.89 months, range: 2.57–50.8 months; *p* = 0.30) (Figure 1). In the preC‐Nfs cohort, the diagnostic delay significantly and inversely correlated with both pNfH and NfL levels in CSF (*r*_s_ = −0.45, 95% CI: −0.64 to −0.20, *p* < 0.001; *r*_s_ = −0.44, 95% CI: −0.63 to −0.19, *p* < 0.001; respectively; Figure 2A). In the C‐Nfs cohort, the diagnostic delay significantly and inversely correlated with CSF NfL levels (*r*_s_ = −0.28, 95% CI: −0.52 to −0.0097, *p* < 0.05), but not with CSF pNfH levels (*r*_s_ = −0.25, 95% CI: −0.49 to 0.028, *p* = 0.07; Figure 2B).

Potential covariates that influence the diagnostic delay were identified in the C‐No Nfs cohort. The diagnostic delay was not shorter in patients with ALS with bulbar onset (*p* = 0.08). Yet, patients carrying a genetic mutation had a significantly shorter diagnostic delay (median: 6.43 months, range: 2.57–32.1) as compared to patients with sporadic ALS (median: 9.97 months, range: 3.03–50.8; *p* < 0.05). The median disease progression rate was equal in all three ALS cohorts (*p* = 0.1, Table 1). Both CSF pNfH and NfL levels significantly correlated with the disease progression rate in the preC‐Nfs cohort (*r*_s_ = 0.29, 95% CI: 0.028–0.52, *p* < 0.05; *r*_s_ = 0.33, 95% CI: 0.069–0.55, *p* < 0.05; respectively) and in the C‐Nfs cohort (*r*_s_ = 0.37, 95% CI: 0.11–0.59, *p* < 0.01; *r*_s_ = 0.40, 95% CI: 0.14–0.61, *p* < 0.01; respectively) (Figure 2C,D). The diagnostic delay significantly and inversely correlated with the disease progression rate in the preC‐Nfs cohort (*r*_s_ = −0.54, 95% CI: −0.71 to −0.33, *p* < 0.0001) and in the C‐Nfs cohort (*r*_s_ = −0.66, 95% CI: −0.79 to −0.47, *p* < 0.0001) (Figure 2E,F).

When the disease progression rate and the presence of a genetic mutation were included as covariates to evaluate the diagnostic delay between the preC‐Nfs cohort and the C‐Nfs cohort, the diagnostic delay was not significantly different anymore between the preC‐Nfs cohort and the C‐Nfs cohort (*p* = 0.14). Furthermore, the median time between the first appointment at the NMRC and the final diagnosis was significantly longer for the C‐Nfs cohort (0 days, range: 0–962 days) as compared to the preC‐No Nfs cohort (median: 0 days, range: 0–46 days, *p* < 0.0001) and the C‐No Nfs cohort (median: 0 days, range: 0–364 days, *p* < 0.0001).

### 3.2 Performance of CSF neurofilaments sampled at early disease stage versus sampled at the NMRC

**FIGURE 3:**
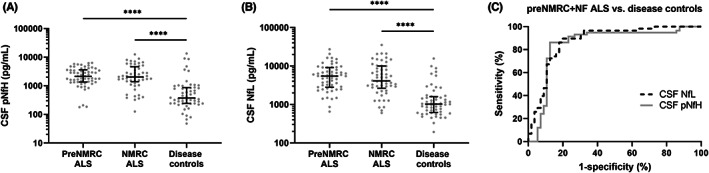
Performance of neurofilament levels at an early disease stage. Scatter plots of phosphorylated neurofilament heavy (pNfH) and neurofilament light (NfL) levels in cerebrospinal fluid (CSF) of patients with amyotrophic lateral sclerosis (ALS) before referral to the neuromuscular reference center (preC‐Nfs), of patients with ALS with Nfs determination at the neuromuscular reference center (C‐Nfs) and of disease controls (A, B). Kruskal–Wallis test with Dunn's post hoc test, ****p < 0.0001. Median and interquartile range are presented on top of the plot. Receiver operating characteristic curves for pNfH (solid line) and NfL levels (dashed line) for the discrimination between preC‐Nfs and disease controls (C).

**FIGURE 4:**
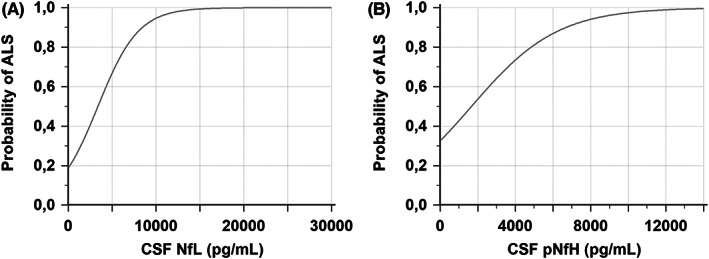
Logistic regression curves to discriminate preC‐Nfs from disease controls. The regression line presents the increasing probability of having amyotrophic lateral sclerosis (ALS) with increasing levels of (A) phosphorylated neurofilament heavy (pNfH) and (B) neurofilament light (NfL) in cerebrospinal fluid (CSF).

Median levels of both pNfH and NfL in CSF were significantly higher in the preC‐Nfs cohort and the C‐Nfs cohort than in the disease control cohort (*p* < 0.0001; Figure 3A,B, Table 1). No significant differences were observed in pNfH and NfL levels between the preC‐Nfs cohort and the C‐No Nfs cohort (Figure 3A,B, Table 1). The area under curve (AUC) of CSF pNfH levels and CSF NfL level to discriminate between preC‐Nfs patients and disease controls was 0.85 (95% CI: 0.77–0.91) and 0.88 (95% CI: 0.80–0.93), respectively (Figure 3C). For CSF pNfH, a cutoff of 1101 pg/ml yielded a sensitivity of 86.2% (95% CI: 74.6–93.9) and a specificity of 87.5% (95% CI: 75.9–94.8%). The positive likelihood ratio was 6.9 (95% CI: 3.4–13.9). For CSF NfL, a cutoff of 1733 pg/ml was associated with a sensitivity of 89.7% (95% CI: 78.8–96.1) and a specificity of 80.4% (95% CI: 67.6–89.8%). The positive likelihood ratio was 4.56 (95% CI: 2.7–7.8). The odds ratio for ALS of pNfH and NfL levels in CSF was 1.0004 (95% CI: 1.0002–1.0007) and 1.0004 (95% CI: 1.0002–1.0006), respectively (Figure 4A,B).

The AUC of CSF pNfH and NfL was 0.84 (95% CI: 0.75–0.90) and 0.85 (95% CI: 0.77–0.91), respectively, to discriminate C‐Nfs from disease controls, and were both not significantly different from the AUC of CSF pNfH (*p* = 0.79) and NfL (*p* = 0.61) determined between preC‐Nfs and disease controls. The sensitivity and specificity of pNfH and NfL levels in CSF to discriminate between C‐Nfs and disease controls were similar to those determined between preC‐Nfs and disease controls (Table 2).

**TABLE 2:** Performance of neurofilaments differentiating patients with ALS from disease controls at time of diagnosis.

	CSF pNfH levels	CSF NfL levels
Cutoff (pg/ml)	>1038	>1682
Area under the curve	0.835 (0.752–0.899)	0.848 (0.767–0.909)
Sensitivity (%)	81.5% (68.6–90.7%)	85.2% (72.9–93.4%)
Specificity (%)	83.9% (71.7–92.4%)	78.6% (65.6–88.4%)
Positive likelihood ratio	5.1 (2.7–9.3)	4.0 (2.4–6.6)

*Note*: 95% confidence interval is provided between brackets.

Abbreviations: CSF, cerebrospinal fluid; NfL, neurofilament light; pNfH, phosphorylated neurofilament heavy.

## 4 DISCUSSION

In this study, we now demonstrate that pNfH and NfL in CSF are significantly increased before diagnosis of patients with mainly sporadic ALS. Yet, the availability of CSF neurofilament values in the early stage did not shorten the diagnostic delay of patients with ALS taken into account other covariates.

Interestingly, without taken into account the covariates, the median diagnostic delay was approximately 3 months shorter in patients with ALS whose neurofilament levels were assessed by a local neurologist before referral to the NMRC (preC‐Nfs) in comparison with patients whose neurofilament levels were only assessed at time of visiting the NMRC (C‐Nfs). No significant difference in diagnostic delay was observed between the preC‐Nfs cohort and patients with ALS who were diagnosed at the NMRC without neurofilament assessment (C‐No Nfs). In the latter group, neurofilament levels were not analyzed probably due to the fact that clinical and electrophysiological examination was sufficient for experienced neurologists to diagnose a patients with ALS. The significantly longer delay between first appointment and final diagnosis observed in the C‐Nfs cohort might indicate that Nfs in CSF are requested by an experienced neurologist in those patients where the diagnosis of ALS is highly uncertain, as reflected by the fact only in 5% of cases riluzole therapy was initiated prior to referral. One could speculate if Nfs had been determined prior to referral in those cases with an uncertain clinical diagnosis at first visit at a NMRC, the delay between first visit at NMRC and final diagnosis might have been shorter.

Local neurologists might be reluctant to diagnose a patient who might have a slow progressive form of ALS as they are not often confronted with such a rare disease.^24^ Indeed, we observed a strong correlation between the disease progression rate and the diagnostic delay. Therefore, in a second step, we did implement this covariate in a model to reassess the diagnostic delay between the preC‐Nfs and the C‐Nfs cohort. We found that the significant difference in diagnostic delay between these cohorts ceased to exist when adding the disease progression rate as a covariate. This indicates that the effect of the disease progression rate on the diagnostic delay might be in imbalance between the two study cohorts. It is known from studies in patients with ALS carrying a genetic mutation that neurofilaments are elevated at an earlier disease stage. Therefore, it might be important to analyze neurofilament levels at an even earlier disease stage, within a prospective study well controlling the disease progression rate in both study arms.^20^, ^21^ Nevertheless, it should be noted that the above‐discussed covariates are often unknown for a local neurologist at such an early disease stage, but if neurofilaments would be analyzed at such a disease stage these biomarkers can be an incentive to persuade local neurologists to diagnose a patient with ALS or to refer them faster to a NMRC. If patients are referred to a NMRC at an early disease stage, it has been shown to shorten the diagnostic delay with almost 4 months.^25^ Moreover, the general practitioner also plays a crucial role to perform additional examinations to quickly refer the patient to a local neurologist and thereby significantly decrease the diagnostic delay in patients with ALS.^26^ Other important factors that might have an potential effect on the diagnostic delay, but have not been addressed in this study, are the regional differences of transport, the coverage of regional neurologists, and the social status of patients visiting the hospital with mild symptoms.

In line with previous findings from our group and others, we strengthened the evidence that levels of pNfH and NfL are significantly increased in CSF of patients with ALS at time of diagnosis in comparison with disease controls.^17^, ^18^, ^19^, ^27^, ^28^ We now explored neurofilament levels in CSF when patients were seen and CSF was sampled by the local neurologist before referral for diagnosis at a NMRC. Interestingly, pNfH and NfL levels were already significantly increased. For CSF NfL, our defined cutoff to discriminate between the preC‐Nfs cohort and disease controls was comparable for NfL at the conventional diagnostic stage at a NMRC. For CSF pNfH, the cutoff increased to 1101 pg/ml to discriminate between the preC‐Nfs cohort and disease controls, as compared to a cutoff of 750 pg/ml at the conventional diagnostic stage at a NMRC.^18^, ^19^ This stage dependent cutoff for pNfH warrants further investigation. This is, however, in line with our findings that pNfH increases in an early stage to ceil at time of diagnosis at a NMRC.^17^ The control cohort included mainly patients with a suspicion of a motor neuron disease, but who in follow‐up were not diagnosed with ALS. These patients were often diagnosed with neurological diseases similar to those of disease mimics in previous studies, that is, inclusion body myositis and radiculopathies.^17^, ^18^, ^19^, ^22^ Another benefit of the disease controls is the fact that these patients were also sampled in a consecutive order by local neurologists similarly to the patients who later on were diagnosed with ALS, providing an unsupervised matched clinical control cohort. Due to the short follow‐up time of the disease controls, a limitation to the study might be that patients are misdiagnosed before obtaining a diagnosis of ALS, as seen by others.^29^

The highest specificity toward motor neuron degeneration was observed for CSF pNfH levels, whereas CSF NfL levels yielded the highest sensitivity. This specific pattern fits into the findings that NfL is rather a global early marker of neurodegeneration, while pNfH is more specific to motor neuron impairment as found previously in CSF sampled at a NMRC.^30^, ^31^ This is exemplified by a case in our disease control cohort concerning a patient with FTD who had normal levels of pNfH (280 pg/ml) but increased levels of NfL (2835 pg/ml), as seen by others.^30^ Therefore, we recommend to measure both neurofilaments, but preferentially pNfH, as diagnostic biomarkers for patients with a suspicion of ALS. Few patients with ALS had low CSF pNfH levels before diagnosis, but the clinical and electrophysiological examinations could have been sufficient for a diagnosis of ALS upon referral. It is not clear yet, at least in CSF, if longitudinal alterations in neurofilament levels serve as potential indicator of a motor neuron disease in early disease stages. We already showed that change in serum pNfH levels is much faster in the early stage of ALS than in other early‐stage neurological diseases.^21^, ^32^

In summary, CSF Nfs assessed before the conventional diagnosis of ALS at a NMRC did not decrease the diagnostic delay when other covariates were taken into account. Given the good discriminating power of Nfs before referral, the added value of Nfs in shortening the diagnostic delay should be further explored prospectively.

## AUTHOR CONTRIBUTIONS

M.D.S., P.V.D., and K.P. developed the concept and design of the study. M.D.S., P.M., P.V.D., and K.P. drafted the manuscript and figures. M.D.S., P.M., P.V.D., and K.P. were involved in the acquisition, analysis, and interpretation of data. Critical revision of the manuscript for important intellectual content was done by all authors.

## FUNDING INFORMATION

M.D.S. has a PhD Fellowship from the Research Foundation—Flanders (11E6319N) and received a grant from the Rotary's “Espoir en Tête – Hoofdzaak er is Hoop.” P.M. has a Research Fellowship from the European Academy of Neurology. K.P. received a Clinical Research fund from the University Hospitals Leuven and is a senior clinical investigator of Research Foundation—Flanders (FWO, Fonds voor Wetenschappelijk Onderzoek Flanders, Belgium). This work was supported by a TBM grant from FWO‐Vlaanderen (no T003519N). P.V.D. is a senior clinical investigator of the Research Foundation—Flanders (FWO, Fonds voor Wetenschappelijk Onderzoek Flanders, Belgium) and is supported through the E. von Behring Chair for Neuromuscular and Neurodegenerative Disorders, the Belgian ALS Liga, and the KU Leuven funds “Een Hart voor ALS,” “Laeversfonds voor ALS Onderzoek,” and the “Valéry Perrier Race against ALS Fund.”

## CONFLICTS OF INTEREST

M.D.S. and P.M. have nothing to report. P.V.D. received a grant from CSL Behring—E. von Behring Chair for Neuromuscular and Neurodegenerative Disorders. P.V.D. participated in advisory board meetings for Biogen, UCB, Cytokinetics, Ferrer, Alexion Pharmaceuticals, Muna Therapeutics, Alector, Argenx, Augustine Therapeutics, QurAlis. P.V.D received honoraria for lectures/presentations from Biogen. K.P. is supported by a grant of the Research Foundation—Flanders (FWO, Fonds voor Wetenschappelijk Onderzoek Flanders, Belgium).

## Supporting information


Table S1

## Data Availability

The data that support the findings of this study are available from the corresponding author upon reasonable request.
