# Supplementary material for: Effect of neurofilament analysis on the diagnostic delay in amyotrophic lateral sclerosis
Source: CNS Neurosci Ther. 2022 Sep 1;29(1):70–7. doi: 10.1111/cns.13960 (PMC9804063; doi:10.1111/cns.13960)
Supplement: Supplementary file 1 — Table S1 [file CNS-29-70-s001.pdf]

Effect of neurofilament analysis on the diagnostic delay in amyotrophic lateral sclerosis

Maxim De Schaepdryver (1), Pegah Masrori (2,3,4), Philip Van Damme (2,3,4), Koen Poesen (1,5)

(1) Laboratory for Molecular Neurobiomarker Research, Department of Neurosciences, Leuven Brain Institute, KU Leuven, Leuven, Belgium.

(2) Laboratory of Neurobiology, Center for Brain & Disease Research, VIB, Leuven, Belgium.

(3) Department of Neurology, University Hospitals Leuven, Leuven, Belgium.

(4) Experimental Neurology, Department of Neurosciences, Leuven Brain Institute, KU Leuven, Leuven, Belgium

(5) Laboratory Medicine, University Hospitals Leuven, Leuven, Belgium.

Supplementary data

Supplementary table 1 Demographics and neurofilament levels of the disease controls

Diagnosis or DD	n	Gender (m/f)	Age	CSF pNfH (pg/mL)	CSF NfL (pg/mL)
Radiculopathy	7	6/1	69 (52-79)	385 (243-8632)	905 (704-8786)
Autoimmune peripheral neuropathy	4	3/1	71 (44-83)	4050 (848-9030)	5982 (1622-7114)
Chronic idiopathic axonal polyneuropathy	4	3/1	76 (72-80)	436 (150-554)	930 (455-1171)
Chronic inflammatory demyelinating polyneuropathy	4	2/2	50 (46-59)	353 (161-790)	718 (566-1733)
Medullopathy	4	3/1	57 (41-68)	439 (217-597)	1051 (647-1332)
Myopathy	3	2/1	56 (50-73)	988 (67-13478)	1540 (452-15738)
Hereditary spastic paraplegia	2	1/1	64 (70-58)	261 (221-301)	705 (561-849)
Inclusion body myositis	2	2/0	81 (76-87)	284 (240-329)	1035 (965-1105)
Plexopathy	2	2/0	55 (50-60)	188 (91-286)	661 (331-991)
Progressive supranuclear palsy	2	1/1	62 (61-62)	2228 (794-3662)	5717 (1506-9928)
Parkinson's disease	2	1/1	72 (61-83)	581 (352-810)	994 (556-1431)
Diabetic polyneuropathy	1	1/0	78	1101	1682
Peripheral nerve entrapment neuropathy	1	1/0	55	195	552
Glioblastoma with gait disorder	1	0/1	71	361	2963
Parkinson's disease and stroke	1	1/0	61	104	1148
Polyneuropathy (toxic/ethylism) and cerebral pathology (posttraumatic intracerebral cyst/vascular LEM)	1	0/1	70	258	768
Peripheral neuropathy	1	1/0	72	293	608

Cramp-fasciculation syndrome	1	1/0	33	48	193
Young-onset dementia	1	1/0	56	238	483
Frontotemporal dementia	1	1/0	75	280	2834
Gait disorder - Spinal lipoma	1	0/1	64	677	1021
Hirayama disease	1	1/0	18	80	450
Myasthenia gravis	1	1/0	81	643	909
Neurosyphilis	1	1/0	51	1449	2749
Paraneoplastic syndrome	1	1/0	74	1035	3341
Parsonage Turner syndrome	1	1/0	61	501	587
Primary lateral sclerosis	1	0/1	72	240	482
Polyarthritis	1	1/0	58	321	1043
Pseudo bulbar palsy	1	0/1	78	1074	1500
Small fiber neuropathy	1	0/1	51	1038	1342
Multiple system atrophy	1	1/0	65	826	2300

Median and range are given. [a]: patient lost to follow-up. CSF: cerebrospinal fluid, pNfH: phosphorylated neurofilament heavy, NfL: neurofilament light, PLS: primary lateral sclerosis, HSP: hereditary spastic paraplegia, MND: motor neuron disease, PSP: progressive supranuclear palsy, LEM: leukoencephalomalacia.

Supplementary table 2 Genetic mutations in the ALS cohorts

	Gene	Mutated	Missing data
**preC-Nfs (n=58)**	*C9orf72*	4 (7%)	4 (7%)
	SOD1	0 (0%)	7 (12%)
	TARDBP	1 (2%)	7 (12%)
	FUS	0 (0%)	7 (12%)
**C-Nfs (n=54)**	*C9orf72*	4 (7%)	0 (0%)
	SOD1	4 (7%)	1 (1%)
	TARDBP	1 (2%)	1 (1%)
	FUS	1 (2%)	1 (1%)
**C-No Nfs (n=180)**	*C9orf72*	17 (9%)	26 (14%)
	SOD1	8 (4%)	45 (25%)
	TARDBP	1 (1%)	47 (26%)
	FUS	0 (0%)	48 (27%)

Genetic status of patients with amyotrophic lateral sclerosis (ALS) with neurofilament (Nfs) assessment before referral to the neuromuscular reference center (preC-Nfs), in patients with ALS with Nfs (C-Nfs) or without NF assessment at the neuromuscular reference center (C-No Nfs). C9orf72: Chromosome 9 open reading frame 72, SOD1: Superoxide dismutase 1, TARDBP: TAR DNA-binding protein, FUS: Fused in sarcoma.
